# Exploring the role of learning goal orientation, instructor reputation, parasocial interaction, and tutor intervention in university students’ MOOC retention: A TAM-TRA based examination

**DOI:** 10.1371/journal.pone.0299014

**Published:** 2024-09-12

**Authors:** Siyao Wang, Sang-Khee Lee

**Affiliations:** Department of Media Communication, Pukyong National University, Busan, Korea; Nanyang Technological University, SINGAPORE

## Abstract

While MOOC platforms allow universities to implement various strategies such as brand promotion and student recruitment, the alarmingly low retention rate suggests a need to explore the critical factors that influence students’ course retention. So far, studies on MOOC platforms focus either on the students’ individual factors (i.e., students’ personal factors such as perceived value) or situational factors (i.e., external influences shaping students’ behavior, such as system quality) for students’ learning, thus lacking a complete view of those determinant factors. This study integrates the TAM model with the TRA model to analyze the roles of three important antecedents (learning goal orientation; LGO, instructor reputation; IR, & parasocial interaction; PI) on university students’ perceived value (PU) and learning attitude (LA), two critical predictors of MOOC retention (CR). Using data from an online survey of 449 Chinese university students, the hypothesis model was tested using PLS. We found that LGO, IR, and PI each positively affect PU; LGO, IR, and PI each positively affect LA; PU and LA each positively influence course retention (CR), with each impact enhanced by tutor intervention (TI). The theoretical and practical implications of such findings are presented.

## Introduction

Digital technologies have empowered the availability of massive open online course (MOOC) platforms such as Coursera, Edx, and Chinese University MOOC. These platforms allow higher education institutions to reconfigure their education models for the purpose of student recruitment, brand promotion, and on-campus learning supplements. The materials on MOOC platforms are available for users to retain, reuse, and redistribute for non-commercial purposes [1]. The open nature and variety of MOOCs have attracted a large number of interested students who seek to overcome barriers in current education by accessing various subjects and learning [2, 3]. However, MOOCs tend to have the issue of low student retention and low completion rates, even for known universities such as Sandford University and MIT [4–6]. Student retention in this study refers to the degree of a MOOC is able to keep students actively enrolled and engaged. Such low retention is attributed to the non-credit-bearing nature of MOOCs and learners’ different motivations (e.g., academic and fulfilling agendas) [7, 8]. However, universities building and introducing MOOCs often aim to enhance students’ knowledge acquisition. Within this context, the low retention rate suggests that university students have not developed knowledge from MOOCs and that universities’ investment in technologies, professors, and funding in MOOCs are underutilized [9, 10] have reminded that the missing physical presence, lack of student involvement, and low completion rates, which could suggest financial losses, learning failures, and even reputation of universities providing MOOCs.

The literature has identified a number of factors that could affect student retention in MOOCs. These factors include students’ individual factors, such as their personal learning habits, motivation, and MOOC learning experience, as well as perceptions of the MOOCs [9]. Among these individual factors, students’ motivation, i.e., an individual’s desire to acquire knowledge from a specific course [11], could significantly affect their retention. Former studies [12–14] suggest motivation to learn as an important antecedent of learning results. As an important motivator, students’ learning goal orientation enables them to seek self-improvement when taking MOOCs. Thus, they are less likely to simply hoard the course materials and avoid the assignments and tests, as some scholars [14, 15] have suggested. However, motivational factors alone may not fully explain students’ course retention. Some researchers [16, 17] suggest that students enrolled in MOOCs are neither engaged nor committed enough. As such, situational factors should be integrated with the motivational variable in explaining students’ MOOC intention. Situational factors refer to the external factors that determine the quality of the learning environment, such as platform design and educators’ support [10]. One important situational factor lies in MOOC instructors’ reputations. Fauth et al. [18] recognize instructor popularity as an important indicator of students’ learning interests and attitudes. Likewise, the Chinese criminal law professor Luo Xiang attracted over five million audiences and followers through online law teaching [19]. While the popularity of MOOC instructors has been well documented in social media, limited academic research has examined the role of instructor popularity in students’ MOOC retention. Moreover, MOOCs often involve a large number of students who socially interact with the instructor through not only online learning platforms but also external social media platforms such as LinkedIn, Twitter, and YouTube [20, 21]. Such interactions may also affect students’ course retention. Indeed, MOOC platforms and social media allow known instructors to appear on different occasions where their personal and academic development could be exposed to inspire the audiences, including students. Studies on the digital environment [22, 23] have identified parasocial interaction, i.e., the face-to-face association between audiences (e.g., students) and performers (e.g., MOOC instructors), in stimulating behavioral responses. While [24, 25] suggest that MOOC platforms should help instructors and students improve student retention through social media, the role of parasocial interaction in the online education context has rarely been examined. Finally, scholars argue that MOOC platform functions such as course video playing, announcement portals, and discussion forums may not effectively facilitate the development of learning communities, which are important for student retention [15]. In this case, universities adopting MOOCs often rely on tutors to implement administrative functions, such as organizing chats, encouraging student participation, and answering students’ logistic questions to ensure pleasant digital learning experiences. So far, not much has been written about students’ perception of tutors’ digital presence in university students’ MOOC retention.

As such, the purpose of this study is to integrate the technology acceptance model with the theory of reasoned action to explore the personal and situational factors that could influence the MOOC retention of university students. As mentioned above, the increasing number of MOOCs provided by universities is confronted with the low rate of student completion. As such, investigating the personal and situational factors not only helps universities to identify the factors when designing MOOCs but, more importantly, helps students to finalize their online learning and facilitate their knowledge acquisition. This study focuses on university students, who, unlike high school students, are given enough freedom to choose courses and constitute the largest market for MOOCs. The objectives of this study involve empirically examining how university students’ learning goal orientation (i.e., personal factor), instructors’ reputation, and student-instructor parasocial interactions influence their perceived usefulness and attitudes towards MOOCs, thereby influencing their course retention, as well as the moderating role of perceived tutor intervention.

This study makes important contributions to literature. First, this study contributes to the growing empirical literature that primarily considers the situational antecedents (i.e., instructor reputation) of digital learning. Specifically, it integrates learners’ individual factors (i.e., learning goal orientation & learning attitudes) from the TRA model to provide a comprehensive view of university students’ MOOC retention. In doing so, this study complements the studies that highlight students’ motivations and perceived value [26, 27].

Second, this study extends the digital learning (e.g., MOOCs) studies [25, 28–31] that highlight the interface design and functionalities of platforms without recognizing the important role of student motivation. While previous studies recognized the personal and situational enablers for students’ course retention, they have not combined those constructs to generate a comprehensive understanding of the differential impacts of each factor. Specifically, it recognizes the lack of instructor-student interaction issue raised by the literature [15, 32, 33] and further suggests parasocial interactions between students and instructors on social media platforms as a possible outlet for the above issue.

Third, this study examines the moderating role of tutor intervention as an important antecedent of university students’ MOOC retention. Few studies have examined how tutors can be appointed to improve students’ MOOC retention by facilitating student-instructor interaction, organizing students’ sense of community, providing suggestions based on instructors’ feedback, and monitoring students’ attendance and learning progress.

The rest part of this paper is organized as follows. First, the literature on TAM and TRA, together with the key variables and their relationship with the dependent variable (i.e., students’ MOOC retention), was discussed, followed by the presentation of the conceptual framework for this study. Next, the research methods will be described, including a discussion of the sample, the variables, and their measurement, as well as reliability and validity. Next, the results are presented, followed by a discussion of the findings, important implications, and limitations. The final section concludes this study.

## Literature review

### A TAM-TRA perspective on student retention

In developing countries such as China, the ambition for mass higher education has driven universities to improve the quality and efficiency of student learning [34]. MOOCs provide a self-paced pattern where students can access recorded lectures and reading materials autonomously, as compared to attending on-site lectures, which are structured by time and space [35]. These courses provide complementary learning opportunities for university students to develop knowledge and professional skills that are missing from their registered programs or universities [36]. MOOCs also improve the exposure of some university professors, some of whom have enjoyed popularity and social media coverage [37]. While MOOCs are widely accepted, the low student retention rate has raised extensive concerns [14, 38], thereby demanding further investigation into the antecedents of student retention of MOOCs.

Most studies on digital learning used the D&M IS Success Model [39], the technology acceptance model (TAM) [40–44] and the theory of reasoned action (TRA) [42] to examine the antecedents of student learning in digital platforms. The D&M IS Success Model considered information quality, system quality, and service quality [39]. However, the model has not considered learners’ involvement in the learning process.

The TAM model includes the factors (e.g., perceived usefulness and one’s subjective perceptions regarding whether technology-based service can bring desirable outcomes [41] that explain the determinants for an individual to adopt or forsake a specific technology-based service. The TRA model considers an individual’s attitude, i.e., one’s assessment of whether performing a behavior could bring positive or negative results [45]. So far, studies on individual adoption of technology-based services have included attitude as an important antecedent [45–48]. In the context of digital learning, Cheung and Vogel [48] proved the impact of attitude on students’ intention to adopt digital learning technology. Similar conclusions were found in several studies [49, 50]. Nevertheless, Li [51] contends that general behavioral models often miss specific situations and thus require context-specific integration. As a result, this study integrates the motivation variable (i.e., learning goal orientation) with three important situational variables, i.e., learning goal orientation, instructor reputation, parasocial interaction, and tutor intervention.

### Students’ learning goal orientation

An individual’s goal orientation could be a predictor of his or her motivation for specific achievements [52], as it can help shape perceptions and regulate behaviors related to the tasks required for a specific goal [53]. In particular, learning goal orientation (LGO) refers to an individual’s efforts and perseverance to acquire new knowledge or develop new skills, with satisfaction by accomplishing a specific task [54, 55]. Learning goal-oriented individuals tend to perceive challenging tasks as opportunities to improve their skills and abilities [56]; in other words, they seek learning opportunities by accepting challenging tasks [31, 52, 57]. When taking MOOCs, students with LGO may aim to understand the new subjects and develop important skills or knowledge that they deem necessary for their study [58]. Previous scholars suggest that LGO as a learning state can be developed in various learning contexts [59], such as MOOC learning [60, 61]. Consumer researchers define perceived value as one’s assessment of whether the functions and performance of a product can meet his or her needs [62]. In the related context of MOOC learning, learning goal-oriented students are likely to recognize the value of MOOCs as they often focus on developing abilities and skills to obtain a sense of accomplishment [63].

Moreover, Albelbisi, Al-Adwan [39] suggested that students need social, technical, and self-management skills to benefit from MOOCs. We further argue that students with a high LGO are more likely to proactively develop those skills. According to [15], students who have accomplished over 20 MOOCs were driven by self-motivated learning and the ambition to accomplish challenging tasks in acquiring more knowledge. In other words, LGO allows students to appreciate and enjoy the challenges of MOOCs. As a result, the following hypothesis can be developed:

**H1:** LGO has a significant positive effect on perceived course value.

Former studies have confirmed that high LGO students are more willing to acquire new knowledge by adapting their attitudes [52]. The knowledge and career development opportunities in social media may attract arts and social science students to develop their computer literacy and digital skills. In particular, learning goal-oriented students may adapt their attitude to video editing, coding, and even programming MOOCs and devote time and effort to learning activities. According to [64], LGO could predict a higher level of learning objectives and superior learning outcomes. In other words, LGO can help shape students’ attitudes toward dedicated learning through MOOCs, allowing them to prepare for the complementary skills that are missing in the current curriculum to prepare for career development. As such, the following hypothesis can be developed:

**H2:** LGO has a significant positive effect on learning attitude.

### Instructor reputation

Early education scholars [65, 66] have recognized the importance of instructor popularity for effective teaching. Students’ favor for a specific instructor could influence their learning in a specific course. This relationship can be explained by the instructors’ various characteristics, such as charismatic teaching style and educational background [67]. Instructors who are known for their academic reputation and engaging and interesting teaching styles are more likely to improve the perceived value of MOOCs. In addition to MOOC platforms, university professors increasingly adopt social media to introduce their academic background and upload short clips to demonstrate various benefits [37, 68]. Such ‘advertisements’ on social media allow students to perceive the values associated with specific MOOCs. Previous studies [32, 66] have provided evidence regarding the role of instructors’ popularity in course retention. In particular, several scholars have identified instructors’ expressive skills and affiliations to be influential to learners’ perceived value [69]. Moreover, instructors with good popularity on social media are more likely to shape students’ attitudes towards specific MOOCs. Given the above discussion, the following hypotheses can be developed:

**H3:** MOOC instructor reputation has a significant positive effect on perceived course value.
**H4:** MOOC instructor reputation has a significant positive effect on learning attitude.

### Student-instructor parasocial interaction

Studies on digital learning platforms such as MOOCs have stressed the importance of instructor-student interaction in students’ attitudes and behavior [15, 32, 70]. In fact, interactions between students and instructors have been recognized as an important determinant of student involvement and engagement in online learning [39]. These scholars suggest that student-instructor interactions and student involvement could enhance students’ learning attitudes. As mentioned above, academics have increasingly adopted social media to publicize their works and research, which attracts tremendous aspiration from audiences [71]. Meanwhile, social media platforms increasingly invite academic celebrities to public speaking [72] and comment on social events. As most universities appoint known academics to deliver MOOCs, those academics are more likely to be recognized by students on social media [72].

While instructors may not be accessible on MOOC platforms, students may maintain parasocial interactions with them on social media platforms. Parasocial interaction refers to the imagined interaction between audiences and social media personalities as if they are interacting with each other in person [73, 74]. Parasocial interaction between audience individuals and media characters like newscasters and actors has been investigated in various research studies [75, 76]. Students who notice the activities of their MOOC instructors on social media may follow those instructors on social media, such as LinkedIn, comment on their latest achievements, and even receive replies. As such parasocial interactions develop, students may perceive the instructors on social media as true friends [77]. The literature suggests that parasocial interactions may change the perceptions of students by helping them realize the different alternatives in their lives [78]. In other words, parasocial interactions may allow students to realize the value of a specific MOOC, e.g., how the related knowledge can be applied in their careers or in society.

According to Katz et al. [79], parasocial interaction can encourage audiences to relate social media celebrities’ messages to their own social experiences. As such, after observing instructors’ introduction to academic background and career development on social media, students may relate such experiences to their own learning and career plans. In doing so, students may realize that the courses mentioned by the instructors may help them realize their own potential for specific roles and jobs. Moreover, cognitive reflections on parasocial interactions may lead to an attitudinal change in the audiences [80]. For instance, students’ attitudes toward MOOCs could change once they develop the perception (through parasocial interactions with instructors on social media) that the MOOCs delivered by the instructor could help them realize specific abilities that lead to the desired outcomes in their lives. Therefore, parasocial interactions with academic celebrities who teach on MOOC platforms may lead to students’ cognitive and attitudinal changes. Drawing on the above discussion, the following hypotheses can be developed:

**H5:** Parasocial interaction has a significant positive effect on perceived course value.
**H6:** Parasocial interaction has a significant positive effect on learning attitude.

### Course retention

The low student retention in MOOCs, reflected in completion rates, has caught tremendous research attention [81]. Recent literature increasingly encourages the exploration of the factors that could influence student retention in MOOCs [32, 82, 83]. Although courses on MOOC platforms are often provided by leading higher education institutions such as Harvard, MIT, and Stanford, the student with an initial interest in obtaining a learning certificate from those institutions may decrease once they realize the efforts required to accomplish a specific course. In this study, we draw on the TAM model to predict that students’ perception of the value associated with a specific MOOC could influence their retention [40]. Former scholars have confirmed how perceived value can affect students’ course retention or continuous use of a technology-based system [44]. As such, the following hypothesis can be developed:

**H7:** Perceived course value has a significant positive effect on course retention.

According to the TRA model, attitude is an important predictor of one’s behavioral intention; it indicates one’s perception of a familiar object to be enjoyable or tedious [83, 84]. Former studies confirmed that students’ attitude toward learning could predict their learning behavior [85]. University students with a learning goal orientation and inspired by instructor reputation and parasocial interaction experience are likely to find joy when taking MOOCs. Such joy could enhance students’ attitudes toward performing specific activities, such as course retention.

As such, the following hypothesis can be developed:

**H8:** Learning attitude has a significant positive effect on course retention.

### Moderating role of tutor intervention

While previous studies stressed the importance of interaction in digital platform learning [86], many MOOC platforms provide limited interaction opportunities between instructors and students. This could discourage students’ need to develop interpersonal relationships and a sense of community. Moreover, insufficient direct communication also prevents students from finding assistance in understanding the various concepts and their applications in a MOOC. Finally, a lack of interaction suggests a lack of discipline that ensures students’ learning progress and discourages their commitment to the course.

Given such problems, universities introducing MOOCs appoint tutors to facilitate student learning [87]. In this case, the tutor plays the surrogate role of an instructor in answering student queries, organizing students’ learning activities and submission of assignments, explaining instructor feedback, providing suggestions, and monitoring students’ progress. These tutors could help students recognize the value of MOOCs and achieve the learning objectives stated in the course descriptions [88]. Moreover, tutors could help students develop a sense of community by forming an e-learning environment with frequent communications, thereby reducing the barriers to distance learning (e.g., watching videos of lectures & answering multiple-choice questions) [15]. In short, the moderation of tutors could enhance the impact of perceived value and learning attitude on students’ course retention. Given the above discussion, we predict the following hypotheses:

**H9:** Tutor intervention positively moderates the relationship between perceived course value and course retention.
**H10:** Tutor intervention positively moderates the relationship between learning attitude and course retention.

**Fig 1:**
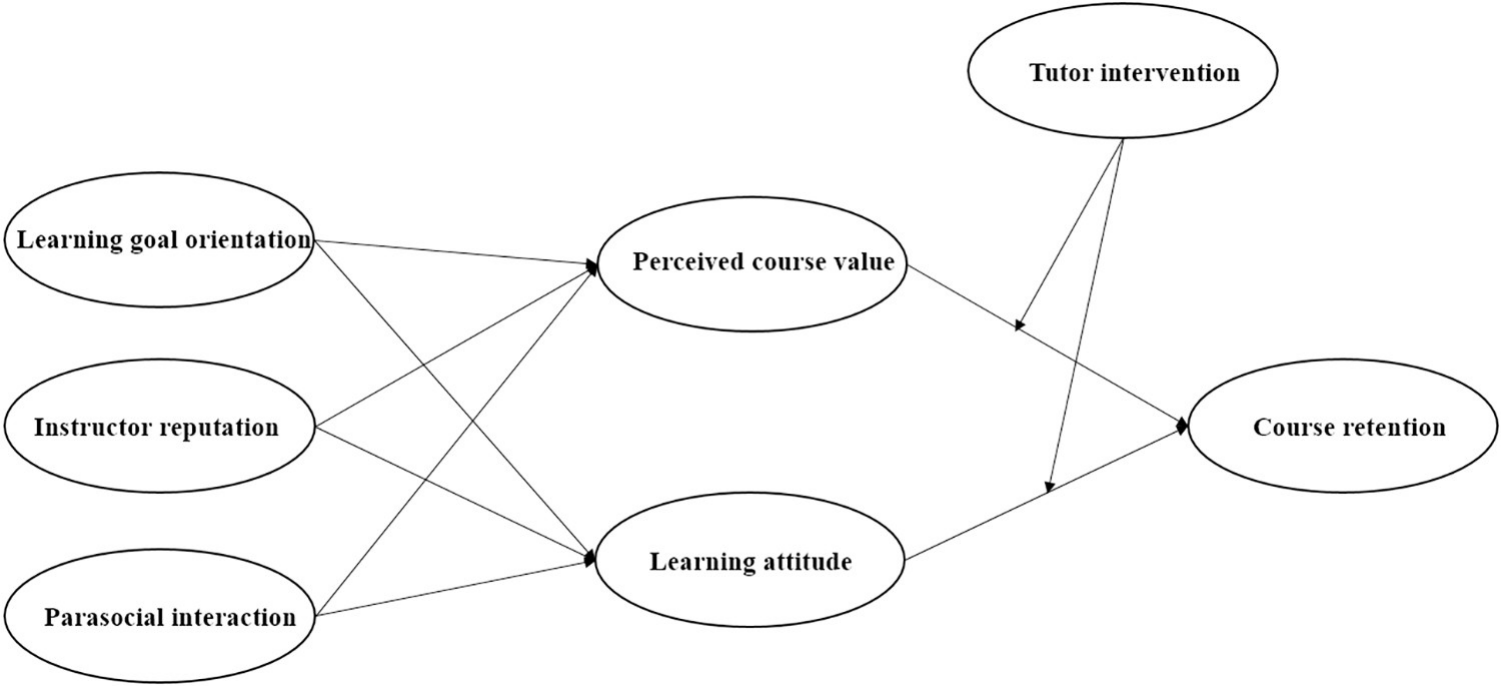
Conceptual model.

The conceptual model is presented in Fig 1.

## Methods

### Sampling

This study aims to examine university students’ MOOC retention. To achieve this objective, a survey was distributed to determine the individual and situational factors that influence university students’ MOOC retention in China. We collected survey data from international universities in southern China. The universities introduced MOOCs from known universities into their library systems and assigned tutors from the libraries to assist students’ MOOC learning. We collected data using the Chinese survey website "WenJuanXing" from October 21st, 2022, to July 21st, 2023. Due to the inaccessibility of all university students’ databases, we adopted convenience sampling techniques to collect data from five universities in Guangdong Province and Zhejiang Province in China. Pukyong National University waived the ethical approval requirement for this study as the study did not have potential risk to the participants and it complied with local legislation and institutional requirements. No one under the age of eighteen participated in this study, and none of the collected data contained sensitive personal information. Respondents were informed of the purpose and instructions for completing the survey. Respondents were permitted to stop answering questions and exit the survey at any time. Respondents were asked to assess their levels of learning goal orientation, instructor reputation, parasocial interaction, perceived course value, learning attitude, tutor intervention, and course retention. The a priori sample size calculator for structural equation models was used to determine the required sample size. Using Free statistics calculators 4.0, anticipated effect size = 0.30 (medium), power level = 0.90, number of latent variables = 7, number of observed variables = 33, and probability = 0.05 were applied, and it was shown that a minimum sample size of 210 was required in the analysis of the current study (Structural equation model). However, the current study included a greater number of subjects to increase the statistical power as well as cope with the possibility of non-response error. We received 465 questionnaires and, after excluding 16 invalid samples, finally received 449 valid samples.

The demographics of the respondents are as follows (Table 1): More than half the respondents were female (N = 244, 54.34%), and 49.67% were social science and humanities majors (N = 223). In terms of education level, 57.46% of the respondents were undergraduates (N = 258), and 42.54% of the respondents were graduates (N = 191).

**Table 1:** Descriptive of demographics.

Demographic items		Frequency	Percentage
**Gender**	Male	205	45.66%
	Female	244	54.34%
**Types of majors**	Natural Science and Engineering	204	45.43%
	Social Sciences and Humanities	223	49.67%
	Sports or Arts	22	4.90%
**Education Level**	undergraduate student	258	57.46%
	graduate student	191	42.54%

### Measures

Since the original scales were created in English, all items underwent a process of back translation [89]. One English-Chinese scholar translated the items into Chinese, and then the other English-Chinese scholar translated them back into English, thereby ensuring an accurate translation. The measurements were graded on a 5-point scale.

### Learning goal orientation

A 5-item scale was adapted from [90] to measure learning goal orientation. Sample items are, “I am willing to select a challenging MOOC that I can learn a lot from,” “I often look for opportunities to develop new skills and knowledge,” and “I enjoy challenging MOOCs where I’ll learn new skills”.

### Instructor reputation

Instructor reputation was measured by a 2-item scale adapted from [91]. Sample items are, “The reputation of the instructor in terms of teaching style was good,” and “The reputation of the instructor in terms of the student’s satisfaction was good”.

### Parasocial interaction

Parasocial interaction was measured by adapting the 4 items from [92]. Sample items are, “I felt free to ask questions from this instructor on social media,” “The instructor responded to my comments and queries,” and “The instructor was easily accessible to me”.

### Perceived course value

Perceived course value was measured by the 4 items adapted from [93]. Sample items are, “The MOOCs I selected would help me to develop career-related skills more quickly,” “The MOOCs I selected would improve my academic performance,” and “The MOOCs I selected would help me prepare for my career in my point of view”.

### Learning attitude

Learning attitude was measured by the 11 items adapted from [94]. Sample items are, “I enjoy finding the solutions by learning from the MOOCs I selected,” “The MOOCs I selected improve my motivation for learning," and "I enjoy the learning experience when taking the MOOCs I selected”.

### Tutor intervention

Tutor intervention was measured by 5 items adapted from [92]. Sample items are, “The tutor played an essential role in facilitating my learning in this course,” “The tutor organized the discussions in this course," and "The tutor was actively helpful when students had problems”.

### Course retention

Course retention was measured by 3 items adapted from [32]. Sample items are, “Did you complete the online courses to earn a credential signifying official completion? (Yes/No). If not, when did you drop out? (First few days, first few weeks, towards the middle, towards the end, just before the end)." "How many exercises/assessments did you complete in the online learning system?" and "How much of the online learning course content do you estimate you watched or read?".

### Data analysis

Smart PLS 4.0 and SPSS 25.0 were used to analyze the data. In order to determine the subjects’ basic level, we first conducted a descriptive analysis. Second, we checked common method bias and evaluated the stability and efficiency of the variables using reliability and validity analysis. Third, we used PLS-SEM to analyze the relationships between the seven variables.

## Results

### Common method bias

To check the problem of common method bias, Harman’s single-factor test was conducted. The analysis returned seven factors with eigenvalues greater than 1, with the first factor explaining less than 40% [95] of the variance (38.76% of 72.41%). Thus, the findings provide no serious indications of common method variance.

### Reliability and validity

For each variable, the test of reliability and validity was conducted (see Table 2). The Cronbach’s alphas ranged from 0.73 to 0.96, consistent with [96], who noted that alpha coefficients above 0.7 are acceptable and those above 0.8 are preferable, indicating the strong attribution relationship between the items and the variables. CR values are all above 0.7, and all AVE values are higher than the suggested 0.5 [97], indicating good convergence validity. The square roots of factors’ AVEs are higher than their correlation coefficients with other factors, which strongly support the discriminant validity [97] presented in Table 3.

**Table 2:** Results of reliability and convergence validity.

Variable	Cronbach’s alpha	Composite reliability	The average variance extracted (AVE)
**CR**	0.82	0.90	0.74
**IR**	0.73	0.88	0.79
**LA**	0.96	0.96	0.70
**LGO**	0.92	0.94	0.75
**PSI**	0.88	0.92	0.74
**PU**	0.87	0.91	0.72
**TI**	0.94	0.92	0.70

Note: CR, Course Retention; IR, Instructor Reputation; LA, Learning Attitude; LGO, Learning Goal Orientation; PIS, Parasocial Interaction; PU, Perceived Course Value; TI, Tutor Intervention. N = 449

**Table 3:** Results of correlations and discriminant validity (Fornell-Larcker criterion).

Constructs	CR	IR	LA	LGO	PSI	PU	TI
**CR**	**0.86**						
**IR**	0.43	**0.89**					
**LA**	0.57	0.56	**0.84**				
**LGO**	0.32	0.23	0.57	**0.86**			
**PSI**	0.46	0.41	0.55	0.29	**0.86**		
**PU**	0.55	0.53	0.54	0.48	0.56	**0.85**	
**TI**	0.09	0.08	0.15	0.07	0.06	0.06	**0.84**

Note: CR, Course Retention; IR, Instructor Reputation; LA, Learning Attitude; LGO, Learning Goal Orientation; PIS, Parasocial Interaction; PU, Perceived Course Usefulness; TI, Tutor Intervention. Values on the diagonal (bolded) represent the square root of AVE, while off-diagonals represent correlations. N = 449

In addition, there is an additional criterion for discriminant validity testing, namely the heterotrait-monotrait ratio of correlations (HTMT), which is illustrated in Table 4. This criterion accepts values less than 0.9 and estimates the correlation between two variables.

**Table 4:** Heterotrait-monotrait ratio (HTMT).

	CR	IR	LA	LGO	PSI	PU	TI
CR							
IR	0.54						
LA	0.63	0.66					
LGO	0.36	0.28	0.60				
PSI	0.54	0.51	0.59	0.31			
PU	0.64	0.66	0.59	0.54	0.64		
TI	0.09	0.08	0.11	0.07	0.04	0.05	

Note: CR, Course Retention; IR, Instructor Reputation; LA, Learning Attitude; LGO, Learning Goal Orientation; PIS, Parasocial Interaction; PU, Perceived Course Value; TI, Tutor Intervention. N = 449

### Collinearity assessment

The values of VIF were computed to check the issue of multi-collinearity in the model. The results of VIF values are between 1.102 and 1.506 (less than 5). Therefore, it is concluded that the issue of multi-collinearity is not present among the variables.

### Structural model

#### Hypothesis testing

**Fig 2:**
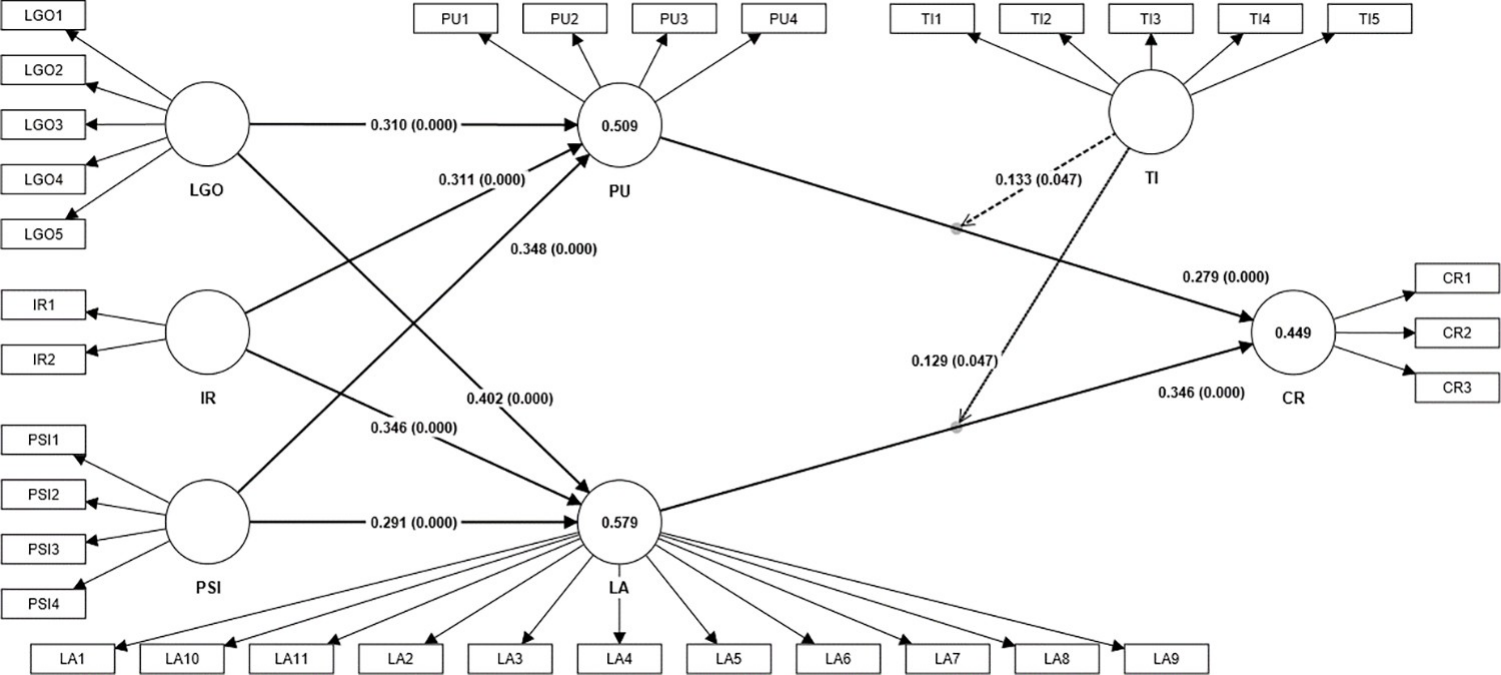
Structural model.

The bootstrapping method (two-tailed test) and the evaluation of T values (significance level p < 0.05 & T value > 1.96) are both used to test the model (see Fig 2). According to the results shown in Table 5, learning goal orientation (β = 0.310, p<0.001), instructor reputation (β = 0.311, p<0.001), and parasocial interaction (β = 0.348, p<0.001) have significant positive effects on perceived course value. As a result, H1, H3, and H5 are supported. Moreover, learning goal orientation (β = 0.402, p<0.001), instructor reputation (β = 0.346, p<0.001), and parasocial interaction (β = 0.291, p<0.001) have significant positive effects on learning attitude. PU (β = 0.279, p<0.001) and LA (β = 0.346, p<0.001) have significant positive effects on course retention. As a result, H2, H4, and H6 are supported.

**Table 5:** Results of hypotheses testing.

Path	β	Standard deviation	T statistics	P values	Results
**IR -> LA**	0.346	0.032	10.819	0.000	Accepted
**IR -> PU**	0.311	0.048	6.507	0.000	Accepted
**LA -> CR**	0.346	0.050	6.910	0.000	Accepted
**LGO -> LA**	0.402	0.031	13.044	0.000	Accepted
**LGO -> PU**	0.310	0.053	5.894	0.000	Accepted
**PSI -> LA**	0.291	0.034	8.619	0.000	Accepted
**PSI -> PU**	0.348	0.061	5.715	0.000	Accepted
**PU -> CR**	0.279	0.055	5.088	0.000	Accepted
**TI -> CR**	0.057	0.072	0.786	0.432	-
**TI x PU -> CR**	0.133	0.067	1.983	0.047	Accepted
**TI x LA -> CR**	0.129	0.065	1.983	0.047	Accepted

Note: CR, Course Retention; IR, Instructor Reputation; LA, Learning Attitude; LGO, Learning Goal Orientation; PIS, Parasocial Interaction; PU, Perceived Course Value; TI, Tutor Intervention. N = 449

**Fig 3:**
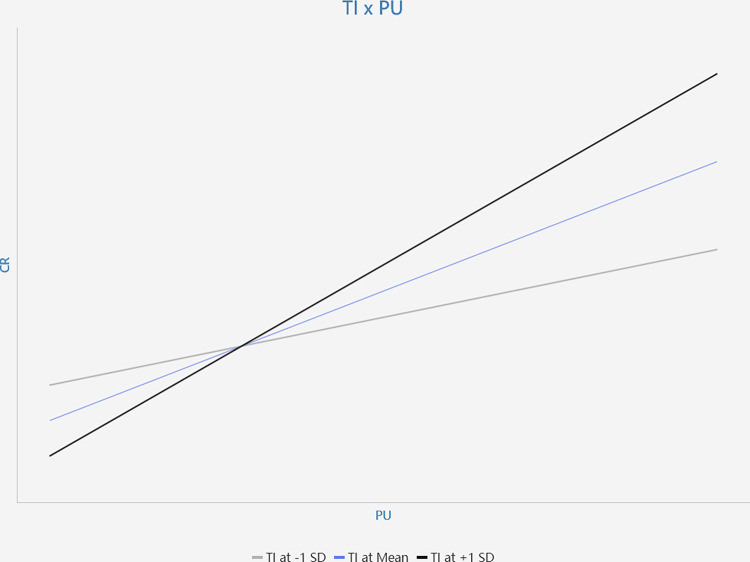
The moderating effect of TI×PU on CR.

**Fig 4:**
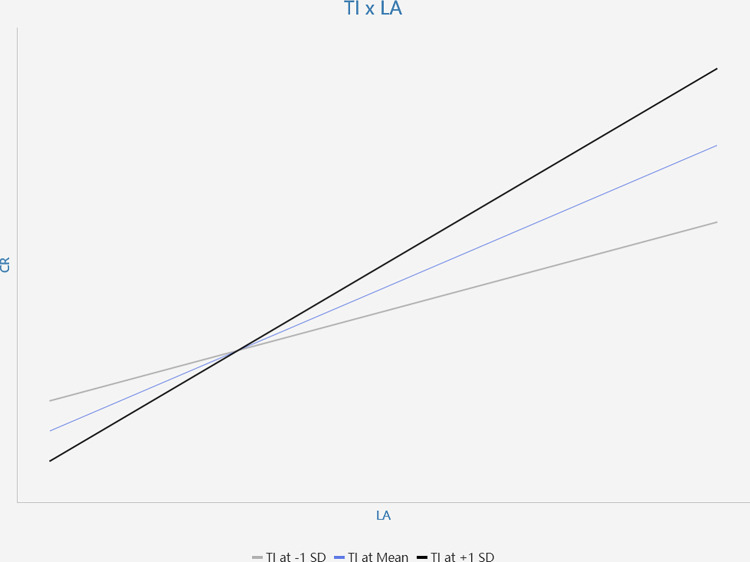
The moderating effect of TI×LA on CR.

The interaction between tutor intervention and perceived course value (TI*PU) has a significant positive effect on course retention (β = 0.133, p<0.05), thereby supporting H9. The interaction between tutor intervention and learning attitude (TI*LA) has a significant positive effect on course retention (β = 0.129, p<0.05), thereby supporting H10. Simple slope plots are presented in Figs 3 and 4. According to these Figures, this study found that the positive relationship of perceived course value (PU) and course retention is stronger when tutor intervention is high; the positive relationship between learning attitude and course retention is stronger when tutor intervention is high. These results further support H9 and H10.

## Discussion

While the educational technology literature has developed increasing knowledge in online learning [4, 62], the surprisingly low rate of completion of MOOCs suggests that more studies are required to understand student retention on MOOC platforms [38]. Integration of student and situational perspectives is important in unravelling how the issues (e.g., poor interaction, low student motivation, & learning material hoarding rather than learning) raised in the literature can be addressed. Therefore, this study aims to identify the factors influencing university students’ MOOC retention. Based on the TAM model and TRA model, we developed and tested a conceptual model to address the research objective. This study was conducted using a random sample of students from international universities in southern China. Data were obtained via an online survey. The current results have been assisted by PLS-SEM analysis.

Our results on the positive impact of learning goal orientation on perceived course value concur with previous studies [63]; this finding suggests that learning goal orientation plays a crucial role in students’ perceived value of taking a specific MOOC. In other words, students are willing to accomplish MOOCs embedded in their university learning platforms because of their motivation to acquire new knowledge and overcome challenges. We also found a significant positive impact of instructor reputation on perceived course value, in contrast to earlier research [65, 66] that investigated the relationship in on-site teaching contexts. Students who feel inspired by academic celebrities on social media could perceive greater value in their selected MOOCs. This study found that parasocial interactions between students and MOOC instructors had a significant positive impact on perceived course value. We also agreed with [64] on the role of learning goal orientation on perceived course value. Students who are learning-goal oriented are more likely to form positive learning attitudes, accept challenges, and appreciate the opportunities for acquiring new skills and knowledge. Our empirical results on the positive role of instructor reputation and parasocial interaction on students’ attitudes towards selected MOOCs. This extends the previous social media [22, 98, 99] studies that confine these two variables in a non-teaching context. Finally, as a new finding, we proposed and empirically confirmed the important role of tutor intervention in enhancing the positive impact of perceived course value and learning attitudes on students’ course retention. This illustrated how perceived course value and learning attitudes are enhanced to improve students’ MOOC course retention.

### Theoretical implication

This study makes several theoretical contributions. First, this study integrates the TAM model with the TRA model, thereby complementing prior studies [42, 44] that primarily focus on perceived value and the situational antecedents (i.e., instructor reputation & parasocial interaction) as the determinant factor when predicting students’ adoption of digital courses. Those situational antecedents include the external factors that influence and shape the conditions for individuals to adopt a specific technology-facilitated course [100]. We included individual factors (i.e., learning goal orientation), thereby extending scholars [42] that primarily focus on external factors. Moreover, we included students’ learning attitudes from the TRA model, thereby tailoring the TAM model according to the requirements of the research context. In doing so, we concur with other scholars’ [51] suggestion to adapt the TAM and TRA models when evaluating the role of key variables in these two models. Drawing on current MOOC studies [15] that raise the issue of students hoarding materials and watching videos as entertainment rather than engaging in learning activities, we explored and examined three crucial factors—learning goal orientation, instructor reputation, and parasocial interaction—and proved that they were significant predictors of MOOC retention. This makes a significant step forward in considering the unique contextual characteristics of MOOC learning not only on MOOC platforms but also on social media platforms, thus recommending course developers and instructors to develop effective strategies to raise the retention rate. Second, in view of the actual challenges of insufficient interactions with instructors, a weak sense of community, and a lack of discipline in the learning process, we examined the moderating effects of tutor intervention. The results demonstrate a missing actor in the MOOC learning process, i.e., course tutors. These tutors could help eliminate the aforementioned barriers, which are innate in MOOC platforms, through timely intervention. Finally, this study was conducted in China, where the COVID-19 quarantine policy lasted until the end of 2022. We shed light on the digital learning literature [42, 68] regarding the introduction of MOOCs during the COVID-19 pandemic to ensure students’ learning.

### Practical implication

Our results also suggest practical implications regarding how the pervasive issue of low student course retention could be addressed. First, given the importance of learning goal orientation, universities introducing MOOCs could first collect students’ learning needs and match these needs with specific courses. Moreover, universities could elaborate on the actual value of introduced MOOCs to stimulate students’ perceived value and shape their learning attitude. While MOOC content is important, students may not be able to appreciate such courses without understanding the associated benefits, course difficulty, and required commitment. Universities could improve students’ learning quality if they import MOOCs based on the understanding of students’ different learning styles and goals to improve engagement and learning experiences.

Second, while MOOCs are based on autonomous learning, students’ expectations for interactions with instructors cannot be neglected. When it is unrealistic to maintain interactions with a large number of students on MOOC platforms, instructors could consider improving their exposure to social media. For instance, instructors could relate their public speeches and academic activities to the MOOCs they are teaching. Doing so could provide students with an additional channel to stay connected with instructors, especially the popular ones, thereby increasing their retention rates.

Third, the findings of this study also suggest that MOOC tutors play an important role in ensuring students’ learning quality and retention on MOOC platforms. For instance, tutors could provide suggestions, based on students’ previous knowledge and experience, on the course suitability, objectives, structure, time and effort commitment, and poke students to keep pace with the required learning progress. Moreover, tutors could allow university students selecting the same courses to meet online and offline, thereby helping them to develop a sense of community. In doing so, MOOC tutors could help address the low completion issue due to a lack of interaction.

### Limitations of the study and future studies

This study only provides a starting point for a better understanding of students’ MOOC retention. We encourage future studies to address some of the limitations of this study. First, we only surveyed university students from China in this study. To better understand and generalize our findings, additional research across regions is encouraged because respondents’ perceptions and attitudes may vary by region. Second, common method bias is a possibility because this study was conducted using a self-reported survey. Even though this study passed the common method bias test, we advise future studies to use longitudinal research or experiments. Furthermore, this study only included student respondents. A comparative study is necessary to determine the differences between students, instructors, and tutors to generalize the MOOC course retention.

## Conclusion

In this study, an integrated TAM-TRA model was utilized to investigate the MOOC retention of Chinese university students. Our ten hypotheses were significantly supported by the empirical data. Specifically, learning goal orientation, instructor reputation, and parasocial interaction can make Chinese university students appreciate the value of MOOCs and shape their learning attitudes toward those courses. Perceived course value and learning attitude accessibility can contribute to university students’ MOOC retention; these relationships are enhanced by tutor moderation.

## Supporting information

S1 Dataset (XLSX)
